# Interest in genetic susceptibility testing and disclosure of AD dementia risk in cognitively normal adults: a survey study

**DOI:** 10.1186/s13195-023-01364-w

**Published:** 2024-01-02

**Authors:** Lisa Waterink, Larissa A. Masselink, Sven J. van der Lee, Leonie N. C. Visser, Solange Cleutjens, Jetske van der Schaar, Argonde C. van Harten, Philip Scheltens, Sietske A. M. Sikkes, Wiesje M. van der Flier, Marissa D. Zwan

**Affiliations:** 1 Alzheimer Center Amsterdam, Neurology, Vrije Universiteit Amsterdam, Amsterdam UMC location VUmc, Boelelaan 1118, 1081 HZ Amsterdam, The Netherlands; 2 Amsterdam Neuroscience, Neurodegeneration, Amsterdam, 1081 HV The Netherlands; 3 Genomics of Neurodegenerative Diseases and Aging, Human Genetics, Vrije Universiteit Amsterdam, Amsterdam UMC location VUmc, Amsterdam, The Netherlands; 4 Division of Clinical Geriatrics, Center for Alzheimer Research, Department of Neurobiology, Care Sciences and Society, Karolinska Institutet, 171 77 Stockholm, Sweden; 5 Department of Medical Psychology, Amsterdam UMC location AMC, University of Amsterdam, Amsterdam, 1105 AZ the Netherlands; 6 Amsterdam Public Health research Institute, Quality of Care, Amsterdam, 1105 BP The Netherlands; 7 EQT Life Sciences Partners, Amsterdam, 1071 DV The Netherlands; 8 Faculty of Behavioural and Movement Sciences, Department of Clinical, Neuro and Developmental Psychology, Vrije Universiteit, Amsterdam, 1081 HV The Netherlands; 9 Department of Epidemiology and Data Science, Amsterdam University Medical Center, Vrije Universiteit Amsterdam, Amsterdam, 1081 HZ The Netherlands

**Keywords:** Genetic screening, APOE disclosure, Dementia risk, Recruitment, Research registry, Clinical trials

## Abstract

**Background:**

Apolipoprotein-E (APOE) genetic testing for Alzheimer’s disease is becoming more important as clinical trials are increasingly targeting individuals carrying APOE-ε4 alleles. Little is known about the interest in finding out one’s genetic risk for Alzheimer’s disease in the general population. Our objective was to examine this in a sample of cognitively normal (CN) adults within a population-based online research registry with the goal to implement APOE-ε4 status for trial recruitment.

**Methods:**

An online survey was completed by 442 CN participants between the age of 49 and 75 years (56% female) from the Dutch Brain Research Registry. The survey assessed interest in participation in research into, and disclosure of, genetic risk for dementia. The survey assessed interest in participation in research into, and disclosure of, genetic risk for dementia and knowing their genetic risk in different hypothetical risk scenarios (10%, 30%, and 50% genetic risk for dementia at age 85, corresponding to APOEε2/ε2 or ε2/ε3, APOEε3/ε4 or ε2ε4, and APOE-ε4/ε4 genotypes). Cochran’s *Q* and post hoc McNemar tests were used to analyse differences in frequencies across scenarios.

**Results:**

Most participants were interested in participating in research into and disclosure of their genetic risk (81%). The most reported reason was to contribute to scientific research (94%). Interest was higher in males, whilst lower-educated participants were more often undecided. When provided with different risk scenarios, interest in knowing their risk was somewhat higher in the scenarios with higher risk, i.e. in the 50% (79%) compared to the 10% scenario (73%;*χ*^2^(2) = 7.98; *p* = .005). Most individuals expected they would share their genetic risk with close relatives (77–89%), would participate in medication trials (79–88%), and would make long-term arrangements, e.g. retirement, health care, will (69–82%), with larger proportions for scenarios with higher hypothetical genetic risk.

**Conclusions:**

Our findings indicate that the vast majority of CN adults participating in a research registry expresses interest in AD genetic risk research and disclosure. Interest in genetic risk disclosure is higher in scenarios corresponding to the APOE-ε4 genotype. This suggests APOE-ε4 screening within an online research registry is potentially a well-received method to accelerate inclusion for trials.

**Supplementary Information:**

The online version contains supplementary material available at 10.1186/s13195-023-01364-w.

## Introduction

Dementia prevalence is steadily increasing worldwide and an important public health issue. Alzheimer’s disease (AD) is the most common cause of dementia and accounts for 50–70% of dementia cases [1]. More research into the prevention of dementia is urgently needed, yet recruitment of research participants for dementia research is difficult, which leads to delays, underpowered studies, or convenience sampling [2]. For prevention trials, it is important to select the appropriate target population, namely individuals at risk for or in the preclinical stages of Alzheimer’s disease. Finding these individuals creates additional challenges [3], because they often do not (yet) experience cognitive impairment and/or information about the presence of AD pathology is lacking. Screening for individuals with increased genetic risk of AD increases the likelihood of finding individuals with AD pathology who might want to participate in prevention trials.

The cause of AD dementia is multifactorial with a considerable genetic component (60–80%) [4]. In the general population, the most important genetic risk factor for AD dementia is the ε4 allele of the apolipoprotein E (APOE) gene [5–8] which is in found roughly 15–25% [9, 10]. This genetic variant increases the lifetime risk of AD dementia by threefold [11, 12], compared to the 15% lifetime risk in the general population. In addition, individuals carrying two APOE-ε4 alleles have an almost 15 times increased risk compared to individuals carrying two APOE-ε3 alleles [10, 13, 14]. Currently, several trials use APOE status as an inclusion criterium in order to increase the likelihood of including participants with AD pathology in cognitively normal adults [15, 16]. However, for effective identification and sizable recruitment of APOE-ε4 carriers, thousands of individuals will need to undergo APOE screening. In addition, screen failures within trials can reach up to 85% [17]. Consequently, screening for prevention trials is a costly and lengthy process, Therefore, improving recruitment and screening methods is a priority within dementia research [17–20].

Online registries can improve recruitment by limiting screen failures to facilitate prescreening for at risk participants. Online registries contain large numbers of voluntary research participants and provide prescreening based on demographic, health, and/or cognitive data. Currently, to our knowledge, a few population-based participant recruitment registries also include genetic information, for example the GeneMatch of the Banner Alzheimer’s Institute [21], the GenePool Study of the Brain Health Registry [22], and the Butler Alzheimer’s Prevention Registry [23]. Both GeneMatch and GenePool do not disclose APOE test results to registrants but only use this information to invite individuals for research participation. However, studies to which the registrants are invited may disclose genetic results as part of the study’s enrolment and screening process. A survey among 25,000 Brain Health Registry registrants showed that the absence of genetic disclosure was not indicated as a barrier to participate in research, but registrants did express high levels of interest in knowing their dementia risk [22].

Previous studies suggest that APOE disclosure can be conducted safely [24–28] and effectively [29]. However, former disclosure studies were mostly performed in specific settings and populations, for instance individuals with first degree family member with AD, or with low levels APOE-ε4 homozygotes and those close to the estimated age of onset. Disclosure within a younger, population-based sample thus remains controversial, and restraint is advised by some due to a lack of proven strategies to prevent or delay disease onset [30]. Recently, the Butler Alzheimer’s Prevention Registry performed APOE genotyping and evaluated the impact of risk disclosure within cognitively normal community dwelling adults and concluded this was safe and well tolerated [23]. They found that APOE-ε4 carriers volunteered more often to screen for prevention trials; however, the number of randomised and enrolled individuals did not differ among carriers and non-carriers. Nevertheless, more knowledge is needed into the interest in genetic susceptibility testing for AD and its impact for cognitively normal individuals within a research context.

Therefore, this study aims to investigate the interest in genetic risk disclosure for dementia within the context of research participation and how this relates to (i) participant characteristics and (ii) different hypothetical risk scenarios corresponding to the APOE genotypes and (iii) the expected impact of risk disclosure among cognitively normal individuals of the Dutch Brain Research Registry (in Dutch: Hersenonderzoek.nl) [31]. The insights obtained will contribute to adequate communication strategies about research participation and genetic risk disclosure, to facilitate implementation of genetic screening within a population-based research registry and ultimately accelerate recruitment for AD dementia research.

## Methods

### Design

We conducted an online, cross-sectional survey among cognitively normal individuals. The Medical Ethics Review Committee of the VU University Medical Center reviewed the study and provided a waiver for ethical approval. All participants provide online informed consent.

### Participants and procedure

We included cognitively normal participants via the Dutch Brain Research Registry (www.hersenonderzoek.nl), a nationwide online registry for participant recruitment for brain disease studies in the Netherlands [31]. The Dutch Brain Research Registry provides information for a lay audience on currently recruiting studies, study results, brain disease-related topics, and information on study participation. Upon subscription, registrants fill out a basic questionnaire about personal, health, and lifestyle information. Based on this information and study-specific inclusion criteria, registrants are found to be eligible and invited to participate in research. For this study, registrants between 49 and 75 years old without a self-reported diagnosis of dementia or mild cognitive impairment (MCI) were eligible. Using purposeful sampling, we invited 1428 registrants via email, aiming for a heterogeneous sample regarding age, gender, education level, and enriched with individuals having first-degree relatives with dementia. In total, 442 registrants participated in the study. Participants’ characteristics and self-reported information about subjective memory complaints and having first-degree relatives with dementia were obtained from the Dutch Brain Research Registry.

### Survey

First, we examined participant’s interest in participation in genetic research and risk disclosure. Possible options included the following: (1) interested in research participation and in risk disclosure, (2) interested in research participation but not in risk disclosure, (3) not interested in research participation nor in risk disclosure, and (4) undecided. Potential reasons for either being interested in risk disclosure or not were rated on a 4-point scale (1 = very unimportant, 2 = unimportant, 3 = important, 4 = very important). Reasons were formulated based on recurring themes in prior research into genetic susceptibility testing of AD [32, 33]. Participants who previously reported to be undecided rated both the reasons for being interested and not being interested in risk disclosure (Table 3). All participants could also provide other reasons in an open text field. As additional participant characteristics, we asked participants to estimate their personal risk for dementia in comparison to the general population (1 = much smaller risk, 2 = smaller risk, 3 = similar risk, 4 = higher risk, 5 = much higher risk).

**Fig. 1:**
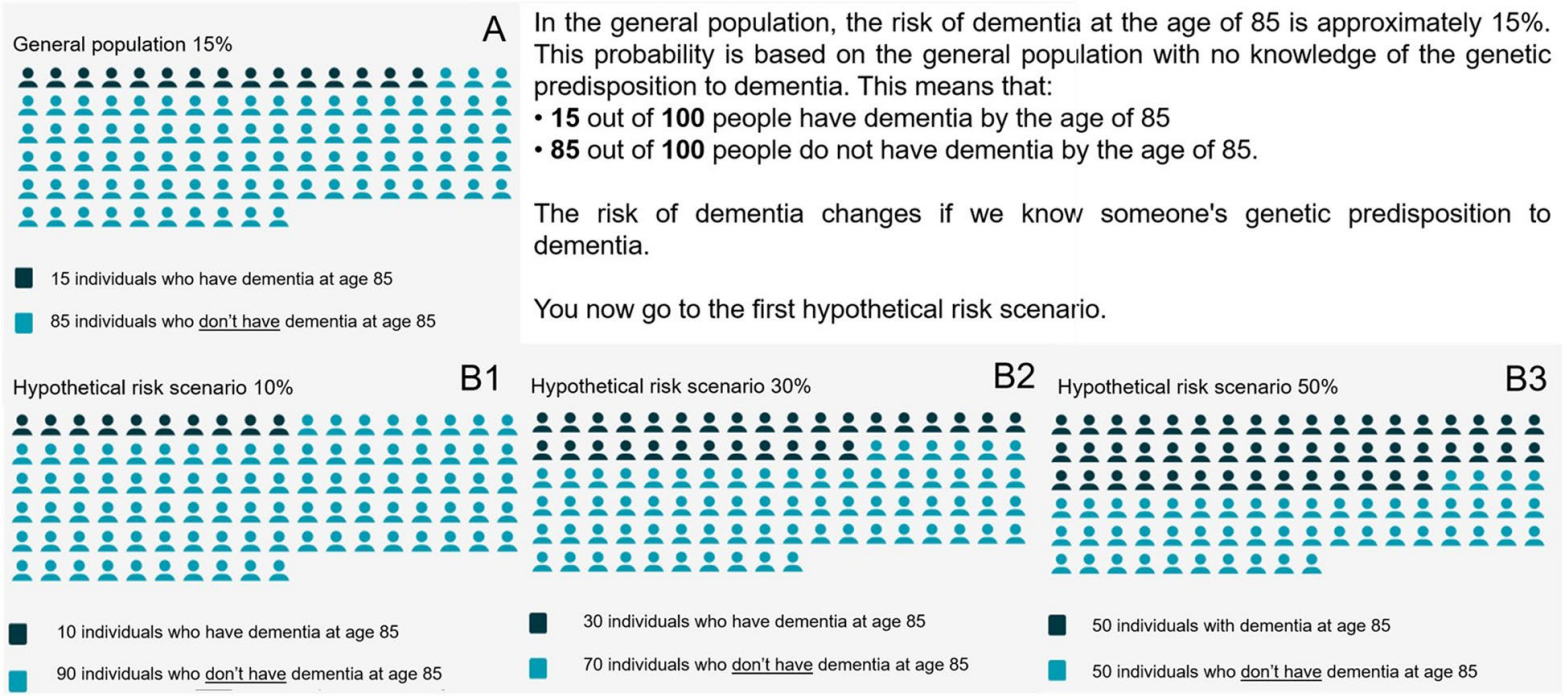
Images used in the survey to present cumulative dementia risk in general population (**A**) and hypothetical risk scenarios (**B1, B2, B3**). Hypothetical risk scenarios B were presented to all participants in random order. After presenting a hypothetical scenario, participants answered the question: ‘If you have this xx% genetic predisposition to dementia, would you like to know?’ and they rated statements about the possible impact (Table 5)

To examine interest in genetic risk disclosure in relation to the APOE genotypes, participants were presented with three hypothetical scenarios, presenting different risks of dementia risk at the age of 85. The APOE-gene and AD dementia were not specifically mentioned in the survey but were referred to as ‘genetic risk factor for dementia’. First, we informed them that the cumulative risk for dementia at the age of 85 in general population is 15%, and after, in random order, we presented scenarios with cumulative risks of 10%, 30%, and 50% (Fig. 1) [34]. These hypothetical scenarios correspond to low risk (ε2ε2 or ε2ε3 genotypes), intermediate risk (ε2ε4 or ε3ε4 genotypes), or high APOE-risk (ε4ε4 genotype) [34]. After reading each scenario, participants were asked if they were interested in knowing their genetic risk (‘If you would have this genetic predisposition (xx%) to dementia, would you like to know?’), and they rated eleven statements about the possible impact of receiving this risk information on a 4-point scale (1 = probably not, 2 = maybe not, 3 = maybe, 4 = probably).

### Data analyses

Participant characteristics (age, gender, education, self-estimated dementia risk, having first-degree relatives with dementia, and subjective memory complaints) and responses to the questionnaire were analysed using descriptive statistics and reported in frequencies. Frequencies are presented in the number and percentage of participants that answered the question (valid percentage). We grouped participants based on their interest in genetic risk disclosure (interested, not interested, and undecided). The relationship between participant characteristics and the interest in risk disclosure was studied using chi-squared tests. Comparisons between groups were calculated with chi-square and the adjusted residuals for which we applied Bonferroni correction for multiple comparison (*p* < 0.006).

For the hypothetical scenarios, to test whether the proportion of participants interested and not interested in risk disclosure (dichotomized by merging not interested and undecided) differed across the different risk scenarios, we performed Cochran’s *Q* tests and post hoc McNemar tests with a Bonferroni correction for multiple comparison (*p* < 0.01). Participants with missing data in any of the scenarios were excluded from the analyses. A sensitivity analysis was performed to see whether participants that not completed the hypothetical scenarios differed from the ones that did. Whether the proportion of participants endorsing possible impact statements of risk disclosure in the scenarios differed was examined with similar methods for which the four-point scale was dichotomized (‘probably not’ merged with ‘maybe not’; ‘maybe’ merged with ‘probably’). We corrected for multiple comparisons with a Bonferroni correction (*p* < 0.002). Data analyses were carried out using the Statistical Package for Social Sciences (SPSS) version 26 (IBM Corp., Armonk, NY).

## Results

### Participant characteristics

Participant characteristics are presented in Table 1. We included *n* = 442 participants with a mean age of 63 ± 7 years of which *n* = 246 (56%) were female. A lower, intermediate, or higher education level was present in respectively 14% (*n* = 63), 49% (*n* = 217), and 37% (*n* = 162) of the participants. Subjective memory complaints were reported by 35% (*n* = 155) and 44% (*n* = 195) had a first-degree relative with dementia. Sixteen participants reported to not know if they had first-degree relatives with dementia. Almost half of the participants (*n* = 214, 49%) estimated they had a similar dementia risk as the general population; 21% (*n* = 94) estimated a (much) lower risk and 30% (*n* = 132) a (much) higher risk (Table 1). Association of participant characteristics with self-estimated risk compared are presented in Supplementary Table 1.

**Table 1:** Participant characteristics

	Sample (*n* = 442)
**Sex**, female (%)	246 (56%)
**Age**, mean (SD)	63 (7)
**Education level**^a^	
Lower	63 (14%)
Intermediate	217 (49%)
Higher	162 (37%)
**Subjective memory complaints**, yes	155 (35%)
**First-degree relatives with dementia,** yes	195 (44%)
**Self-estimated dementia risk**^b^	
Smaller risk	94 (21%)
Similar risk	214 (48%)
Higher risk	132 (30%)

Results are presented in % of total participants; subjective memory complaints missing *n* = 3; having first-degree relatives with dementia ‘unknown’ *n* = 16. Self-estimated dementia risk missing *n* = 2

^a^Lower = primary school, lower level of secondary school or lower vocational training. Intermediate = higher level of secondary school or intermediate vocational training. Higher = higher vocational training, university or academic education

^b^Compared to the general population

### Interest in research participation and genetic risk disclosure for dementia

**Fig. 2:**
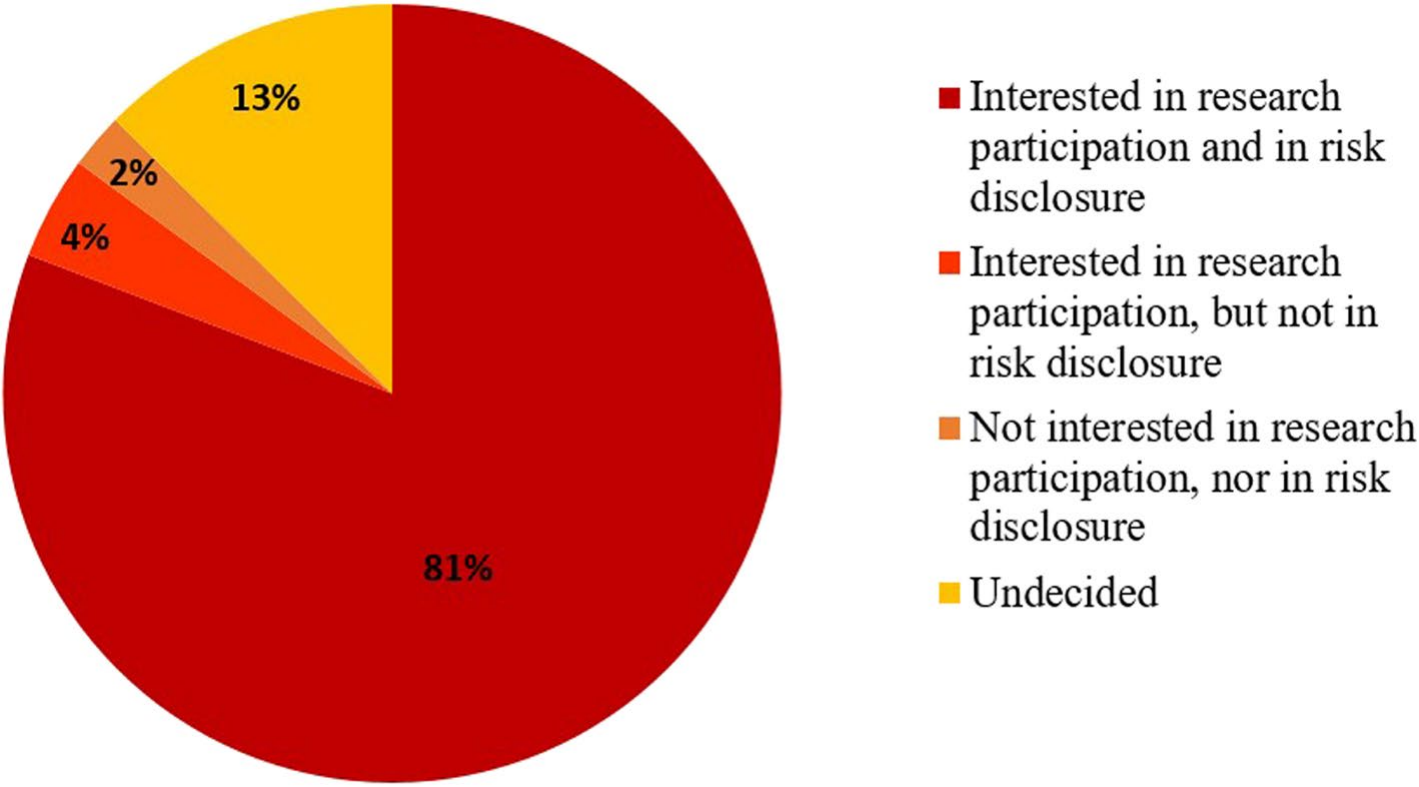
Frequencies of interest in participation in genetic research and risk disclosure. Results are presented in % of total valid; missing *n* = 5

Figure 2 shows that most participants (*n* = 353, 81%) were interested in both research participation and genetic risk disclosure, whilst 13% (*n* = 55) was left undecided. A small proportion (*n* = 29, 6%) was not interested in genetic risk disclosure, of which one third was not interested in research participation.

### Relationship between interest in genetic risk disclosure and participant characteristics

Males and highly educated participants were more often interested in research participation and genetic risk disclosure (both *p* < 0.05; Table 2). Participants with lower education were more often undecided (*p = .*036*)*. Interest in risk disclosure was not related to subjective memory complaints, having a family member with dementia, or self-estimated risk for dementia.

**Table 2:** Associations between participant characteristics and interest in genetic risk disclosure for dementia

	Interested (*n* = 353)	Not interested (*n* = 29)	Undecided (*n* = 55)	Overall *p*-value
**Age** ≥ 63	199 (57%)	19 (66%)	31 (57%)	.630
**Sex**, female	185 (52%)^b^	22 (76%)^c^	37 (67%)	**.009**
**Education level**				
Lower	45 (13%)	3 (10%)	12 (22%)^d^	**.030**
Intermediate	168 (48%)	14 (48%)	33 (60%)	
Higher	140 (40%)^e^	12 (41%)	10 (18%)^f^	
**Subjective memory complaints**, yes	125 (36%)	9 (32%)	19 (35%)	.927
**First-degree relatives with dementia**, yes	158 (45%)	11 (38%)	24 (44%)	.288
**Self-estimated dementia risk**^a^				.466
Lower risk	73 (21%)	6 (21%)	15 (27%)	
Similar risk	168 (48%)	17 (58%)	27 (49%)	
Higher risk	112 (32%)	6 (21%)	13 (24%)	

Results are presented in number of participants (% of total valid). Subjective memory complaints, missing *n =* 3; first-degree relatives with dementia, ‘unknown’ *n =* 16

^a^Compared to the general population. Differences were tested using chi-square. Post hoc analysis, after Bonferroni correction for multiple comparisons (*p* < 0.006), showed difference with: ^b^less often reported compared to men; ^c^more often reported compared to men; ^d^more often reported compared to higher educated; ^e^more often reported then other groups; ^f^less often reported then other groups. Significant levels are presented in bold

In Table 3, all the reasons that were presented in the survey for either being interested or not interested in the result of their genetic risk of dementia are listed including the number and percentages of participants endorsing these reasons. The most important motivation for being interested in risk disclosure was ‘I want to contribute to scientific research’; 94% (*n* = 381) found this (very) important, followed by 87% (*n* = 354) endorsing ‘I want to know my genetic risk’, and 71% (*n* = 290) endorsing ‘My genetic risk might give me information about the risk for my children/relatives’ (Table 3). Other reasons in favour of disclosing genetic risk mentioned were (1) to prepare for the future by arranging personal affairs and inform family (e.g. euthanasia, care and housing) (*n* = 18, 4%) for example ‘to be able to anticipate in a timely manner in terms of housing, and possibly care, if necessary’ and (2) to prevent or slow progression with medication/lifestyle changes (*n* = 12, 3%) for example ‘to be able to intervene earlier in the event of symptoms arising’.

**Table 3:** Number of participants endorsing reasons for either being interested or not interested in the result of their genetic risk of dementia

	(Very) important
**Reasons for interest in risk disclosure** (*n* = 406)	
1. I want to contribute to scientific research	381 (94%)
2. I want to know my genetic risk	354 (87%)
3. My genetic risk might give me information about the risk for my children/family members	290 (71%)
**Reasons for no interest in risk disclosure** (*n* = 80)	
1. I would be very worried if I had an increased risk of developing dementia	69 (86%)
2. Dementia currently cannot be prevented or treated	60 (75%)
3. The test will not give me absolute certainty that I will develop dementia	54 (68%)
4. I do not want to know my genetic risk	50 (63%)
5. My genetic risk might give me information about the risk for my children/family members, and I do not want to know this	40 (50%)
6. I am afraid of negative social consequences	34 (43%)

Results are presented as the number of participants (%) out of the total number participants that were presented with the statements; missing *n* = 6. Participants that were undecided rated all reasons

In those not interested in risk disclosure (*n* = 29, 6%,) and those who were undecided (*n* = 55, 13%), the most common reasons for not wanting to know were ‘I would be very worried if I had an increased risk for developing dementia’ (*n* = 69, 86%), ‘Dementia currently cannot be prevented or treated’ (*n* = 60, 75%), and ‘The test will not give me absolute certainty that I will develop dementia’ (*n* = 54, 68%). One other reason that was mentioned by one participant was ‘knowing to have increased risk of dementia could possibly have consequences for my mortgage and insurances’.

### Hypothetical risk scenarios and impact statements

When presented the hypothetical risk scenarios corresponding to the APOE genotypes, interest in risk disclosure was generally common with more than 70% of the participants being interested across scenarios. Nonetheless, interest varied across scenarios (*n* = 409; *χ*^2^_Cochran’s*Q*_ (2) = 10.93; *p* < .004; Table 4), as significantly higher proportion of participants are interested in disclosure in the 50% scenario (*n* = 330, 79%) compared to the 10% scenario (*n* = 308, 73%; post hoc analysis *χ*^2^_McNemar_(2) = 7.89; *p* = .005). No significant difference was found in the proportion of interested participants in risk disclosure between the 10% and 30% scenarios nor between the 30% and 50% scenarios. Participants who did not answer all hypothetical scenarios (one or more scenario missing) were lower educated, more often reported memory complaints, and less often had a first-degree family member with dementia (see Supplementary Table 3).

**Table 4:** Interest in genetic risk disclosure of dementia in three hypothetical risk scenarios (10, 30, or 50% risk of dementia at the age of 85)

	10% scenario (*n* = 423)	30% scenario (*n* = 416)	50% scenario (*n* = 417)
Interested	308 (73%)	321 (77%)	330 (79%)
Undecided	63 (15%)	61 (15%)	58 (14%)
Not interested	52 (12%)	34 (8%)	29 (7%)

Results are presented in number of participants (% of total valid); 10% scenario, missing *n* = 19; 30% scenario, missing *n* = 26; 50% scenario, missing *n* =25. Hypothetical risk scenarios 10%, 30%, and 50% presented in random order to all participants. For analysis, not interested and undecided were merged. Post hoc McNemar test showed a difference after Bonferroni correction for multiple comparisons (*p* < 0.01) between scenarios: interest in the 50% scenario was higher compared to 10% risk scenario

Finally, we asked how the information on (increased) genetic risk would impact participants’ behaviour, by providing them with eleven statements on possible impact after each hypothetical risk scenario. The three most endorsed impact statements of risk disclosure across scenarios were (1) ‘I would share my genetic risk with my close relatives’ (77–89%); (2) ‘I would participate in medication trials’ (79–88%); and (3) ‘I would make long-term arrangements, e.g. retirement, health care, will’ (69–82%; Table 5). Most participants disagreed with the impact statement ‘I would choose a less healthy lifestyle’ (86%–90%). The proportion of participants endorsing the impact statements differed between hypothetical scenarios, i.e. the expected impact increased when the dementia risk increased (*p* < .002), except for the statement ‘I would choose a less healthy lifestyle’ (*p* = .045; Table 5). Post hoc analysis showed that all proportions on the impact statements increased compared to the 10% scenario (*p* < 0.002). When comparing the 30% and 50% genetic risk scenarios, the proportions endorsing the following two statements also differed ‘I would be worried about my risk of dementia’ (73% versus 80%) and ‘I would feel sad’ (37% versus 49%) (both *p* < 0.002; Table 5 and Supplementary Table 3). Sensitivity analysis showed that participants who did not answer all hypothetical scenarios were more often lower educated, had subjective memory complaints, and less often had a first-degree relative with dementia (Supplementary Table 2).

**Table 5:** Percentage of participants endorsing impact statements

Possible impact after receiving genetic risk, *n* (%)	10% scenario	30% scenario	50% scenario	Overall *p*-value
1. I would participate in medication trials	331 (79%)	355 (87%)^a^	363 (88%)^a^	< **.001**
2. I would share my genetic risk with my close relatives	322 (77%)	351 (86%)^a^	369 (89%)^a^	< **.001**
3. I would make long-term arrangements (e.g. retirement, health care, will)	289 (69%)	322 (79%)^a^	339 (82%)^a^	< **.001**
4. I would be mentally more active (e.g. starting a new hobby or making puzzles)	230 (57%)	272 (66%)^a^	278 (67%)^a^	< **.001**
5. I would exercise more	225 (54%)	260 (63%)^a^	277 (67%)^a^	< **.001**
6. I would eat healthier	215 (51%)	250 (61%)^a^	267 (65%)^a^	< **.001**
7. I would sooner do the things I’ve always wanted	214 (51%)	271 (66%)^a^	284 (69%)^a^	< **.001**
8. I would be worried about my risk of dementia	189 (45%)	298 (73%)^a^	332 (80%)^a, b^	< **.001**
9. I would be worried about the risk of dementia for my children or family members	170 (43%)	257 (66%)^a^	279 (71%)^a^	< **.001**
10. I would feel sad	99 (25%)	147 (37%)^a^	194 (49%)^a, b^	< **.001**
11. I would choose a less healthy lifestyle	39 (10%)	53 (14%)	52 (13%)	.045

All participants rated the 11 impact statements after being presented with each of the three hypothetical risk scenarios, on a 4-point scale (1 = probably not, 2 = maybe not, 3 = maybe, 4 = probably). Results presented in this table are numbers (% of total valid) of participants that rated the statement with a 3 or a 4. 10% scenario, missing *n* = 19; 30% scenario, missing *n* = 26; 50% scenario, missing *n* =25). Overall, Cochran’s *Q* showed differences between scenarios in expected impact regarding 10 out of the 11 statements (*p*-values < .001). Post hoc McNemar test showed the following differences between scenarios, after Bonferroni correction for multiple comparisons (*p*-values < 0.002): ^a^more often endorsed compared to 10% risk scenario; ^b^more often endorsed compared to 30% risk scenario (see Supplementary Table 3). Significant *p*-values are presented in bold

## Discussion

Our most important finding was that the vast majority (81%) of cognitively normal participants recruited from a population-based brain research registry wish to know their genetic risk for developing dementia when participating in genetic research. When presented with three hypothetical dementia risk scenarios corresponding to different APOE genotypes, participants were more interested in genetic risk disclosure and more likely to participate in medication trials, when the presented likelihood of developing dementia was higher. APOE-ε4 screening within an online research registry thus seems to be a well-received method to facilitate recruitment of individuals carrying APOE-ε4 genotypes for preclinical trials that include the disclosure of genetic risk to participants.

Our finding of a high interest in genetic risk disclosure is in line with previous studies in both individuals at risk for AD dementia [35, 36] and in cognitively normal elderly [37–39]. Most frequently endorsed reasons for interest were to contribute to scientific research but also to inform themselves and their relatives about their genetic risk for dementia and make long-term arrangements, as similar to other studies [40, 41]. It is not surprising that we found a high willingness for research participation and genetic disclosure among research volunteers of a brain research registry, as these individuals may be more interested in personal health-related information. Nonetheless, our sample also examined the attitudes of participants without family members with dementia of which less is known, and showed similar levels of interest

In line with previous findings, we found that males are more interested in risk disclosure than females [36, 42]. We did not confirm the previously reported association with age [36, 43, 44]. In our study, interest in risk disclosure was also related to education, as higher educated individuals were more often interested and lower educated individuals more often undecided. Other studies also found an increased interest in higher-educated individuals [37]; however, others showed opposite association [38]. Variations in results may be due to differences in age or specific settings and populations. Nonetheless, these results give direction for tailored educational tools and dissemination about risk disclosure and research participation. Additionally, emphasising the importance of this matter are the results of the sensitivity analysis revealing that participants abstaining to answer the hypothetical scenarios were lower educated, more often had subjective memory problems, and less often had a family member with dementia. Tailored educational tools and dissemination could for example consist of videos with detailed explanation about genetic risk and possible impact and use language that is appropriate at B1 proficiency to abate uncertainties about information provision and enable a well-informed decision.

Interest in genetic risk disclosure was high in all hypothetical scenarios corresponding to different APOE genotypes (73–79%). Previous studies showed that expressed interest in risk disclosure exceeds actual participation in disclosure. For instance, only 24% of adult children of AD patients with an interest in risk disclosure progressed to actual disclosure [45], which was comparable to results of genetic resting for Huntington disease in the 1980s [46–48]. Nonetheless, participants’ interest in risk disclosure was somewhat higher in response to scenarios in which their hypothetical risk was higher. We expected the lower risk scenario the be more favourable, because of the higher chance to rule out dementia. However, our findings indicate that higher genetic risk is especially relevant for individuals to know, despite the uncertainty and larger personal impact. Possible explanation of interest in risk disclosure in all scenarios could be due to selection bias. Research volunteers within the Dutch Brain Research Registry, specifically the ones participating within this study, would probably have high interest in genetic susceptibility testing independent of risk scenarios. An additional explanation could be that participants did not perceive the low-risk scenario (10%, corresponding to the ε2ε2 or ε2ε3 genotypes) as significantly or meaningfully lower than the presented general population risk (15%). It must be noted that the concept of risk is generally difficult to comprehend and we did not verify how the hypothetical scenarios were understood. Nevertheless, Roberts et al. (2000) also found that the pros of risk disclosure outweighed the cons for many individuals and that individuals might underestimate the limitations and risks of genetic testing and disclosure [49].

Previous studies found that disclosure does not lead to significant depression or anxiety symptoms in APOE-ε4 carriers in the short term, both in controlled research trials [24, 25, 50] and in direct-to-consumer testing [51]. Hypothetical scenarios in the present study suggested that genetic risk disclosure can trigger feelings of worry and sadness. Surprisingly, we found this both for the low-risk scenario (corresponding to ε2ε2 or ε2ε3 genotypes) and (to a greater extent) in the high-risk scenario (corresponding to ε4ε4 genotype). This finding may be due to prior worries about dementia which we did not take into account in this study. Previous studies showed that anxious and depressive feelings prior to genetic counselling could be predictive for increase in psychological distress after disclosure. However, the sense of relieve, control, and positive behavioural changes after learning the results might outweigh the adverse psychological reactions [52]. Additionally, previous disclosure studies emphasize the importance of guided counselling to reduce dementia concerns [27]. Another explanation might be that a proportion of participants did not fully grasp the hypothetical scenarios and results about adverse psychological reactions might be an overestimation.

On the other hand, disclosing APOE genotype may have a positive impact in terms of health and lifestyle changes, even after being informed none of these changes were proven to prevent AD dementia [37, 53]. In the current study, we found that more than half of participants indicated that if they were to find out they had an increased dementia risk, they would adopt positive lifestyle changes (‘I would be mentally more active/exercise more/eat healthier.’). However, actual behavioural changes after genetic disclosure are expected to be different from behavioural intentions expressed in response to hypothetical scenarios. It is known that changing behaviour is difficult because of complex interplay between intrapersonal and external factors like motivation, behavioural capacity, and self-efficacy [54]. Moreover, endorsed behavioural changes may be associated with our specific study sample. As mentioned above, individuals in the Dutch Brain Research Registry may be more engaged with their personal health. In line with this, a study of Christensen et al. (2015) showed that differences in behavioural changes after genetic disclosure were related to different recruitment strategies [55]. So, implications from this study and other genetic disclosure studies may not apply to populations that are less prone to proactively seek out genetic susceptibility testing. However, our results do underscore that a large number of individuals wish to know their genetic risk and want to take preventive actions, for example participate in clinical trials or change health and lifestyle behaviour [56]. This could be due to the growing awareness of the relationship between healthy living and dementia risk reduction [57–59].

Younger age, having first-degree relatives with dementia, and the presence of subjective memory complaints were associated with a higher self-estimated dementia risk compared to the general population but not with interest in risk disclosure. One in three participants believed their personal risk was higher than the general population risk, which could be explained by the large proportion of participants with first-degree relatives with dementia and subjective memory complaints. Slooter et al. (1998) estimated that 25% of the general population aged 55 years and older have a first-degree relative with dementia, compared to 44% within our study [60]. ‘Dementia runs in the family’ or ‘I have memory complaints’ were also the most endorsed reasons for higher self-estimated risk. However, self-estimated high dementia risk was not related to interest in risk disclosure. This indicates that motives for risk disclosure are not exclusively depended on one’s dementia risk perception and that reasons can be very personal.

Commercially available direct-to-consumer genetic screening tests including APOE genotype have become more widely available, and the interest among the general population in genetic susceptibly testing increases [51, 61, 62]. With the current increase in interest in commercially available APOE-genetic screening tests [45], like 23andMe, and associated requests to explain genetic results [58], and growing demand of personalised dementia risk reduction [56], the need for accurate education about genetic risk and disclosure impact arises. Historically, genetic disclosure was assigned to medical doctors providing education and information about impact. This is however neither efficient nor scalable for a research setting. Previous participant registries have successfully used (remote) APOE genotyping as screening to recruit participants [21, 22] and provided frameworks for scalable genetic counselling and (telephonic) disclosure within a research context [29, 63]. However, more research is needed to align APOE-genetic disclosure protocols within a research setting for cognitively normal adults without first degree relatives with dementia [64]. Currently, evidence about safe disclosure to cognitively normal research volunteers is emerging [23, 64]. Our study provides additional evidence of the generally positive attitudes of cognitively normal adults towards genetic screening for research purposes, and underscores the high interest in disclosure.

## Strengths and limitations

This study had several limitations; the present sample was selected from the Dutch Brain Research Registry [31], in which participants have registered because they are potentially interested to participate in brain research and genetic disclosure which limits the generalisability of our findings to the general population. However, our large sample provided the opportunity to study the effect of multiple participant characteristics (age, gender, education level, having first-degree relatives with dementia) on the interest in risk disclosure. Secondly, participant selection from a research registry provided us with a representative of individuals who want to participate in scientific research or prevention trials. Unfortunately, we were not able include information about social-economic status or racial background. We acknowledge the importance to diversify the samples enrolling in genetic susceptibility testing research, to generalise the findings to the general population. Another possible limitation is the online nature of the questionnaire, as we could not control whether participants interpreted the questions correctly, and hypothetical scenarios were understood. Additionally, the present study investigated interest in risk disclosure using hypothetical research participation and personal risk scenarios. It is therefore important that future studies examine actual participation in genetic risk disclosure and its long-term impact in a real-life setting.

## Conclusion

In conclusion, this study showed that most cognitively normal adults enlisted in a research registry are interested in research participation and disclosure of genetic dementia risk. Future studies are needed to provide more insights into the actual impact and attitudes after research participation and genetic risk disclosure for dementia, ethical aspects, and ways to provide appropriate counselling to enable a well-informed decision and minimise the psychological burden on member of a population-based research registry. The next step would be to include genetic screening within the Dutch Brain Research Registry to optimise recruitment for clinical trial and research studies.

### Supplementary Information

**Additional file 1: Supplementary Table 1.** Self-estimated dementia risk in comparison to the general population in association with participant characteristics. **Supplementary Table 2.** Sensitivity analysis comparing participants that completed all hypothetical scenarios and those who did not answer all scenarios. **Supplementary Table 3.** Results of post-hoc testing McNemar for the eleven impact statements.

## Data Availability

The datasets generated and/or analysed during the current study are not publicly available due to ethical reasons since the subject did not provide consent for data sharing.
