# Supplementary material for: Interest in genetic susceptibility testing and disclosure of AD dementia risk in cognitively normal adults: a survey study
Source: Alzheimers Res Ther. 2024 Jan 2;16:1. doi: 10.1186/s13195-023-01364-w (PMC10759504; doi:10.1186/s13195-023-01364-w)
Supplement: Supplementary file 1 — Additional file 1: Supplementary Table 1. Self-estimated dementia risk in comparison to the general population in association with participant characteristics. Supplementary Table 2. Sensitivity analysis comparing participants that completed all hypothetical scenarios and those who did not answer all scenarios. Supplementary Table 3. Results of post-hoc testing McNemar for the eleven impact statements. [file 13195_2023_1364_MOESM1_ESM.docx]

**Supplementary Table 1.** Self-estimated dementia risk in comparison to the general population in association with participant characteristics.

|  | **Lower risk**  **(n=94)** | **Similar risk**  **(n=214)** | **Higher risk**  **(n=132)** | **Chi-square**  **p-value** |
| --- | --- | --- | --- | --- |
| **Age** >63 | 57 (61%) | 135 (63%) | 59 (45%) | **<.001** |
| **Sex,** male | 47 (50%) | 88 (41%) | 60 (46%) | .335 |
| **Education level**  Lower  Intermediate  Higher | 11 (12%)  40 (42%)  43 (46%)^*^ | 39 (18%)  116 (54%)  60 (28%)^†^ | 13 (10%)  60 (46%)  59 (45%)^‡^ | **.004** |
| **Subjective memory complaints**, yes | 20 (21%) | 88 (41%) | 47 (36%) | **.003** |
| **First-degree relatives with dementia**, yes | 16 (17%) | 80 (37%) | 98 (74%) | **<.001** |

Notes. Post-hoc analysis showed:  ^*^More often reported similar chance compared to other groups. ^†^Less often reported compared to other groups ^‡^More often reported compared to lower education. Significant levels are presented in bold.

**Supplementary Table 2.** Sensitivity analysis comparing participants that completed all hypothetical scenarios and those who did not answer all scenarios.

|  | **Complete**  **(n=409)** | **Missing**  **(n=33)** | **Overall**  **p-value** |
| --- | --- | --- | --- |
| **Age** ≥ 63 | 233 (45%) | 18 (36%) | .787 |
| **Sex,** female | 225 (55%) | 21 (64%) | .337 |
| **Education level**  Lower  Intermediate  Higher | 51 (13%)  198 (48%)  160 (39%) | 12 (36%)^†^  19 (58%)  2 (6%) | **<.001** |
| **Subjective memory complaints**, yes | 135 (33%) | 20 (61%) | **.002** |
| **First-degree relatives with dementia**, yes | 189 (46%) | 6 (18%) | **.004** |
| **Self –estimated dementia risk***  Lower risk  Similar risk  Higher risk | 45 (18%)  138 (55%)  66 (27%) | 7 (23%)  17 (55%)  7 (23%) | .792 |

Notes. Results are presented in number of participants (% of total valid). Subjective memory complaints, missing *n =* 3; First-degree relatives with dementia, ‘unknown’ *n =* 16.
^*^ Compared to the general population. Differences were tested using chi-square. Post-hoc analysis, after Bonferroni correction for multiple comparisons (p< 0.008), showed difference with: ^†^more often lower educated compared to higher educated

**Supplementary Table 3.** Results of post-hoc testing McNemar for the eleven impact statements.

| **Possible impact after receiving genetic risk**, n (%) | **10% vs. 30% scenario** | **30% vs. 50% scenario** | **10 vs. 50% scenario** |
| --- | --- | --- | --- |
| 1. I would participate in medication trials | **<.001** | .454 | **<.001** |
| 1. I would share my genetic risk with my close relatives | **<.001** | .018 | **<.001** |
| 1. I would make long-term arrangements (e.g. retirement, health care, testament) | **<.001** | .015 | **<.001** |
| 1. I would be mentally more active (e.g. starting a new hobby or making puzzles) | **<.001** | .710 | **<.001** |
| 1. I would exercise more | **<.001** | .020 | **<.001** |
| 1. I would eat healthier | **<.001** | .037 | **<.001** |
| 1. I would sooner do the things I’ve always wanted | **<.001** | .155 | **<.001** |
| 1. I would be worried about my risk of dementia | **<.001** | **<.001** | **<.001** |
| 1. I would be worried about the risk of dementia for my children or family members | **<.001** | .003 | **<.001** |
| 1. I would feel sad | **<.001** | **<.001** | **<.001** |
| 1. I would choose a less healthy lifestyle | .025 | 1.00 | .090 |

Notes. All participants rated the 11 impact statements after being presented with each of the three hypothetical risk scenarios, on a 4-point scale (1 = probably not, 2 = maybe not, 3 = maybe, 4 = probably). Results presented in this table are numbers (% of total valid) of participants that rated the statement with a 3 or a 4. 10% scenario, missing *n* = 19; 30% scenario, missing *n* = 26; 50% scenario, missing *n* =25). Significant levels are presented in bold, after correction for multiple comparisons (p <0.002).
